# Aligned Matching: Improving Small Object Detection in SSD

**DOI:** 10.3390/s23052589

**Published:** 2023-02-26

**Authors:** Seok-Hoon Kang, Joon-Sang Park

**Affiliations:** Department of Computer Engineering, Hongik University, Mapo-gu, Seoul 04066, Republic of Korea

**Keywords:** convolutional neural network, object detection, small object, single shot multibox detector, matching strategy

## Abstract

Although detecting small objects is critical in various applications, neural network models designed and trained for generic object detection struggle to do so with precision. For example, the popular Single Shot MultiBox Detector (SSD) tends to perform poorly for small objects, and balancing the performance of SSD across different sized objects remains challenging. In this study, we argue that the current IoU-based matching strategy used in SSD reduces the training efficiency for small objects due to improper matches between default boxes and ground truth objects. To address this issue and improve the performance of SSD in detecting small objects, we propose a new matching strategy called aligned matching that considers aspect ratios and center-point distance in addition to IoU. The results of experiments on the TT100K and Pascal VOC datasets show that SSD with aligned matching detected small objects significantly better without sacrificing performance on large objects or requiring extra parameters.

## 1. Introduction

The use of convolutional neural networks (CNNs) is a key technique in various machine vision tasks such as object detection, image segmentation, and action detection [1,2,3,4,5,6]. Although several transformer-based object detectors such as DETR [7] and SWIN [8] have recently been developed, they are computationally demanding and still less efficient than CNN models in terms of both data and time [9]. CNN-based object detection schemes can be divided into two types, including single- and two-stage detectors. Two-stage detectors such as R-CNN [1] and Faster R-CNN [2] perform a localization task first and then perform detection and classification in the target areas. Because two-stage detectors use a separate network for each task, they typically achieve high accuracy and are widely applied for tasks that require precision. However, this performance comes at the cost of a relatively long inference time and a high number of parameters. In contrast, single-stage detectors such as Single Shot MultiBox Detector [4] (SSD) and You Only Look Once [3] (YOLO) perform localization and classification tasks simultaneously. Hence, single-stage detectors can perform inferences rapidly and require relatively few parameters. However, they also exhibit lower accuracy.

Single-stage detectors are widely used in tasks that require real-time object detection, such as intelligent video surveillance and autonomous driving. However, they are often unsuitable for practical applications due to their low accuracy. In autonomous driving, for example, detectors must be able to identify not only large objects such as adjacent cars but also small objects such as traffic signs and signals. However, this remains a difficult problem in practice, because detecting small objects is a computationally challenging task.

Several methods have been developed to solve this problem. Krisantal et al. [10] proposed a data augmentation technique for small object detection. By oversampling images containing small objects and augmenting them with multiple instances of small objects extracted using segmented masks, their method achieved enhanced performance on small object detection tasks. However, this augmentation process does involve an increased computational demand.

A tiling approach was investigated in Ref. [11]. In other words, when the resolution of the input images was higher than the input layer of the CNN model being used, each input image was divided into smaller tiles to train the network. This approach can prevent the loss of detail on small objects in input images. This technique is useful when system memory is limited such that the CNN model cannot be scaled up to the resolution of the input images. Although this method showed an improvement in accuracy, it requires processing a large number of tiles of input images, which inevitably increases the training and inference time accordingly.

Using a multi-scale feature map for detection has also been shown to improve small object detection performance. SSD was designed to detect both small and large objects using multi-scale feature maps [4]. To detect objects at multiple scales, SSD uses multiple feature maps with different scales and applies a classifier and localizer to them rather than using a single feature map in the last layer. SSD also uses default boxes similar to Fast R-CNN’s anchor boxes. Default (bounding) boxes are associated with each cell of a feature map to detect objects relative to their shape. Although SSD is faster and more accurate than contemporary two-stage detectors, it also involves the disadvantage of relatively poor performance for small objects. Feature Pyramid Network [12] (FPN) models were developed to utilize not only bottom-up but also top-down pathways. The FPN first passes an input image to a bottom-up pathway, which produces feature maps with smaller sizes and stronger features. The feature map of the last layer is then is upsampled through a top-down pathway. These upsampled features are enhanced by features from the bottom-up pathway via lateral connections. Thus, FPN models can generate high-resolution feature maps with strong features from deep layers, which provides an advantage for the localization of small objects. This simple and effective structure of FPN models can extract information about objects of all scales. Hence, numerous subsequent works have adopted this approach [13,14,15]. As an application of FPN models, Deconvolutional Single Shot Detector [16] (DSSD) has also been developed to improve on the structure of SSD by stacking deconvolution layers at the back of the extra layer of SSD. Similar to FPN models, DSSD utilizes lateral connections with the front layer to fuse feature maps with the most detailed information lost in the bottom most layer. Additionally, by using a ResNet [17] backbone and replacing the prediction layer with a residual block, DSSD is also designed to take advantage of skip connections to provide feature maps with more semantic information. However, because these additional connections increase computational costs, this model can be slower than the original SSD. Similarly, CAB Net [18] employing multiple parallel branches (or pipelines) of multi-scale feature maps with different dilation rates, sacrificed the inference speed for the enhanced small object detection performance.

Enabling the model to observe only certain parts of an input more deeply, which is referred to as attention, has also been shown to help enhance the performance of small-object detection methods [7,19,20]. Attention and Feature Fusion SSD [21] (AF-SSD) improved the SSD for small-object detection by introducing three modules, including feature fusion (FFM), dual-path attention (DAM), and multiscale receptive field (MRF). To suppress the background noise, spatial and channel-wise attention is used in DAM. To deal with the lack of semantic information in shallow layers, FFM builds top-down pathways to fuse semantic information from deeper layers to shallower layers, similar to FPN models. MRF performs convolutions in parallel with various sizes of receptive fields. Using these three modules, AF-SSD was able to increase detection performance, especially for small objects. However, this design also requires a larger model size and perform inferences more slowly.

Customizing the training strategy for small-object detection could be an effective approach. SNIP [22] aimed to train a network model efficiently by backpropagating only the gradient of an object instance within an acceptable scale range. Although SNIP dramatically improved detection performance at various object scales, it also slowed down the training process. Subsequently, Singh et al. proposed SNIPER [23], which is another strategy for multi-scale training. Instead of processing an entire image pyramid, it selectively processed context regions surrounding ground truth instances at the proper scale. To expedite multi-scale training, SNIPER sampled low-resolution regions from a multi-scale image pyramid.

As shown in ATSS [24], the label assignment process can significantly influence the performance of a detector. In anchor-based detectors, regulating the intersection over union (IoU) threshold based on which positive and negative samples are labeled is among the simplest ways to enhance the effectiveness of label assignment. Lowering the IoU threshold makes matching easier for small objects. However, this may result in selecting an anchor box that is distant from the ground truth object. ATSS used statistical methods for choosing positive and negative samples because the IoU distribution between the samples and ground truth boxes resembled the normal distribution. PAA [25] used the Gaussian Mixture Model (GMM) of two modalities to model the distribution of scores indicating whether an anchor is positive or negative. The problems of using IoU threshold-based strategies in the label assignment in multi-stage detectors (e.g., Faster R-CNN) have been discussed and alternatives to IoU such as Gaussian receptive field have been proposed in Refs. [26,27].

In this study, we argue that the matching strategy in the training process of SSD can be enhanced, and the performance of SSD in detecting small objects can be enhanced using our improved matching strategy without requiring any additional parameters or computational resources for training. In our matching strategy, called aligned matching, each ground truth data sample is matched to a default box by considering not only the IoU value but also the distance and aspect ratio similarity between them, whereas the matching in SSD is primarily based only on the IoU value. Our method helps increase the training efficiency of SSD by finding better matches between default boxes and ground truth objects. We present the results of an experimental investigation to show that the performance improvement of SSD with our proposed approach can be dramatic in some cases, e.g., a 60% increase in mAP. The source code and trained weight files used in this study are available at https://github.com/ksh108405/aligned-matching (accessed on 26 January 2023).

The remainder of this study is organized as follows. In Section 2, we first discuss the matching strategy used in the training process of SSD and then discuss our proposed approach, called aligned matching, in Section 3. We present the results of an experimental evaluation of our proposed approach in Section 4 and conclude the work with a summary of our findings in Section 5.

## 2. Problems with Training SSD on Small Objects

One straightforward method to enhance the performance of SSD for small-object detection tasks is to increase the size of the input. Increasing the input size weakens the degree of downsampling of the input image, allowing for more detailed information about the target object to be received. To validate this proposition, we investigated the performance of SSD on a dataset focused on small objects called Tsinghua-Tencent 100K [28]. We used a PyTorch implementation of SSD [29] with the minimum default box size of 0.1. Table 1 shows the performance of SSD with different input sizes of 300 × 300 (SSD300), 512 × 512 (SSD512), and 1024 × 1024 (SSD1024) on the dataset. Five classes (pn, pne, i5, p11, pl40) that appear the most in the dataset were used for training (80%) and evaluation (20%). Notably, with a minimum default box size of 0.1, the results show that the performance of the SSD1024 model was 23.4% in terms of mAP, 30.5% lower than that of the SSD512 model, which was 33.7% mAP. These counterintuitive results suggest that problems other than those arising from low-resolution input inhibit SSD from learning to detect small objects. We argue that the main causes of this problem lie in the ground truth-default box *matching strategy* in SSD’s training process.

The original SSD network uses a truncated VGG-16 network as a base, followed by additional feature layers. Because these extra feature layers progressively decrease in size, SSD can perform detections at multiple scales. Detection is performed by applying predictor layers to these multiple feature layers (or maps). Each cell on each feature map is assigned six default boxes with aspect ratios of 1, 2, 3, 1/2, or 1/3. The size (or scale) of a default box depends on the size of a feature map to which it belongs. The smallest default boxes are associated with the lowest layer feature map, termed conv4_3, which is part of the base VGG-16 network. Their size is a performance parameter of SSD that users can change, along with the largest size of the default boxes. The sizes of the remaining default boxes are to be determined using an equation. That is, the size of *k*th smallest default boxes is calculated as $s_{k}=s_{\min}+\frac{s_{\max}-s_{\min}}{m-1}\left(k-1\right),\;k\in \left[1,m\right]$ where *m* is the number of feature maps, *s*_min_ is the smallest size of the default boxes, and *s*_max_ is the largest size.

In the training process of SSD, each ground truth box is matched to a default box based on the best intersection over union (IoU) value [4]. However, this strategy may not work well when the ground truth is too small such that it is completely covered by the areas of multiple default boxes. This strategy may reduce the training efficiency for small objects for two reasons. First, a ground truth object may not be located near the center of the receptive field of the selected default box. Each pixel in the receptive field of a CNN layer can have a different degree of impact on the output. In fact, the distribution of impact within the receptive field is asymptotically Gaussian [30]. Thus, it is important that the ground truth should be located at the center of the receptive field to avoid diminishing the efficiency of the training process. Second, the aspect ratio of a ground truth object can be disregarded. Because a set of default boxes of the same area with different types of aspect ratios is considered, multiple default boxes with different aspect ratios can show the same highest IoU. As a result, a ground truth sample may be matched to a default box with different aspect ratios. Moreover, floating-point arithmetic involving irrational numbers, such as the width or height of a default box being a multiple of $\sqrt{2}$, can lead to imprecise IoU calculations as in most SSD implementations.

**Figure 1:**
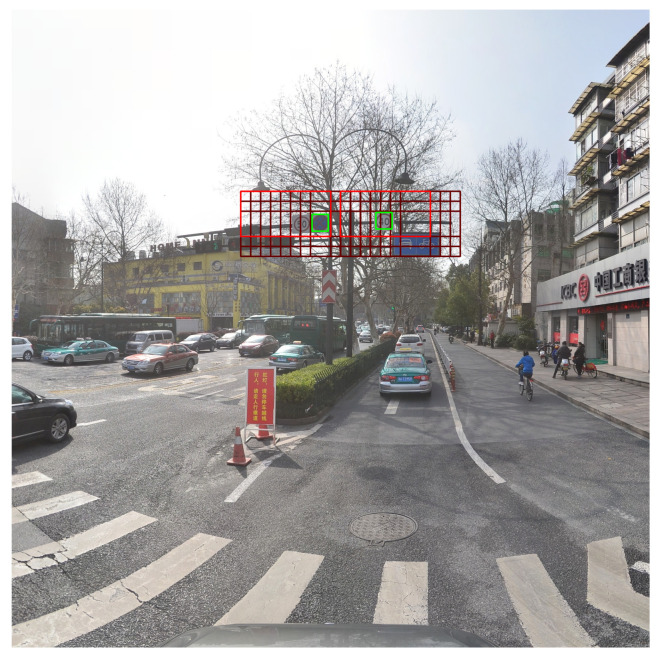
Example of problematic matching in SSD training with TT100K [28]. (Green boxes represent ground-truth objects, red boxes are the default boxes matched to the objects, and dark red boxes are the default boxes with the same IoU as the matched default boxes).

Typical problematic ground truth-default box matching cases are visualized in Figure 1. It may be observed in the figure that small ground truth objects (green boxes) are fully included in the areas of several default boxes (dark red boxes) with the same highest IoU value. In these cases, each ground truth object is matched to a default box (red box) such that the ground truth object is located at the corner or bottom of the matched default box. This is because when there are multiple default boxes with the same IoU, a randomly selected box becomes a match. Moreover, owing to the IoU only criterion, default boxes with 1:2 aspect ratio are matched to the ground truth objects whose aspect ratios are near 1:1.

Overall, SSD’s matching strategy may not work as intended for small objects. This mainly occurs because SSD’s matching strategy relies solely on IoU and there is lack of a fallback option when multiple default boxes have the same IoU. If the matching strategy fails to locate a ground truth box at the center of a receptive field, the training efficiency is diminished. This is because within a receptive field, each pixel’s impact on the output is distributed asymptotically as Gaussian and decaying rapidly from the center [30], as mentioned previously. Since the number of default boxes increases with the input size of the SSD network model, the problem is expected to worsen in SSD network models with larger input sizes. In such cases, there is a higher probability of matching the ground truth with an inappropriate default box. The SSD1024 model’s low performance can be explained in this context.

**Figure 2:**
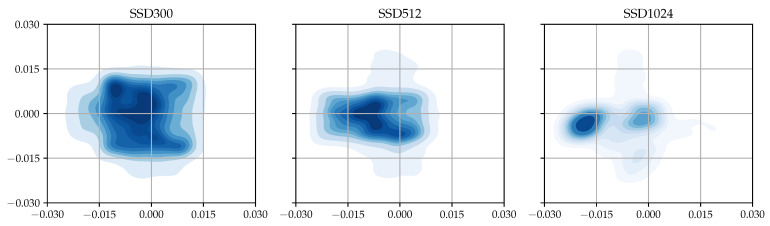
Relative center point distribution obtained from the TT100K dataset.

By comparing the positions of the center points of a ground truth object and the default box matched to it, we can determine how often such an inappropriate matching occurs when training SSD with the TT100K dataset. To this end, we plotted all coordinates of the center points of the default boxes relative to the center points of their matched ground truth boxes in Figure 2 as a 2D histogram. All the relative positions between each ground truth’s center point and the center point of the default box matched to the ground truth were collected during training SSD with the TT100K dataset. The darker the color of an area, the higher the density of points. As shown in the figure, for example, the center points of ground truth objects tended to move farther from the center points of their matched default boxes with an increasing input size. In the case of SSD with 1024 × 1024 input, the position of the majority of ground truth objects diverged from the center of their matched default boxes, that is, a wide scattered area with the darkest region located relatively far from the center may be observed.

This problem can be alleviated by optimizing the size of the default boxes. However, this involves two pitfalls. Changing the scales of default boxes has an impact on the overall performance of SSD. This might lead to lower performance for tasks of detecting objects of diverse sizes. Additionally, the scales of the default boxes must be optimized for a specific dataset along with the network input size, which may involve a series of repeated training processes. Alternatively, K-means clustering, which groups data into K clusters while minimizing variances within the clusters, can be applied to optimize the sizes of default boxes as in YOLO. The K-means clustering technique can provide appropriate default box sizes such that the objects in the training set are clustered based on their sizes and the default box sizes are set to the clusters’ centroids. However, this approach may show degraded performance when the distribution of objects’ sizes varies between the training and test sets.

## 3. Aligned Matching

In this section, we introduce a new matching strategy referred to as aligned matching to alleviate the aforementioned problems in SSD’s matching strategy. Pseudocode describing the proposed method is presented in Algorithm 1.
**Algorithm 1** Aligned Matching **Input:** Ground truth *t*, Default boxes $D=\{d_{1},d_{2},...,d_{n}\}$ **Output:** Matched Default box *d*_matched_1:max_iou ← 02:**for** $d_{i}\in D$ **do**3:    **if** max_iou < IoU$\left(t,d_{i}\right)$ **then**        ▹ Calculate IoU for each default box4:        max_iou ← IoU$\left(t,d_{i}\right)$                 ▹ Find maximal IoU5:**for** $d_{i}\in D$ **do**6:    **if not** IoU$(t,d_{i})>$ max_iou **then**7:        $D\leftarrow D-\{d_{i}\}$        ▹ Discard default boxes wtih non-maximal IoUs8:**for** $d_{i}\in D$ **do**9:    **if** ratio_class(t)≠ ratio_class$\left(d_{i}\right)$ **then**  ▹ ratio_class(x) returns the ratio class of *x*10:        $D\leftarrow D-\{d_{i}\}$               ▹ Check if ratio class is different11:min_dist ←∞12:**for** $d_{i}\in D$ **do**13:    **if** min_dist > distance$\left(t,d_{i}\right)$ **then**     ▹ Calculate distance for each default box14:        min_dist ← distance$\left(t,d_{i}\right)$15:        *d*_matched_$\leftarrow d_{i}$                ▹ Set result to nearest default box

To find a match to the ground truth, the algorithm filters out the default boxes in *D* stepwise using different criteria. We apply the maximal IoU criteria: calculate IoU for each default box, find the maximal IoU value, and then exclude from *D* default boxes with non-maximal IoUs. Assuming that IoU values are calculated using floating-point arithmetic, the calculations are rounded up to six decimal places to disregard small variances in IoU. This is necessary in most SSD implementations because floating-point arithmetic involving irrational numbers, e.g., the width of a default box being a multiple of $\sqrt{2}$, can lead to imprecise IoU calculations. Second, an aspect ratio similarity criterion is applied. That is, default boxes with aspect ratios that differ from that of the ground truth are excluded. The ratio class of x is defined as the x’s aspect ratio rounded up to the closest number in {1,2,1/2,3,1/3}, which are the five types of aspect ratios that default boxes can have. By comparing the ratio classes of the remaining default boxes in *D* with that of the ground truth box, the algorithm deletes the default boxes that do not correspond to the ground truth in terms of their aspect ratios. Finally, among the default boxes stored in *D* satisfying these criteria, the one with the closest distance to the ground truth is selected. The distance between a default box and a ground truth box is the Euclidean distance between their center points. If there are multiple default boxes that have the same distance to the ground truth remaining in *D*, which is generally quite unlikely, a random selection is made. Again, our approach considers not only IoU but also the aspect ratio similarity and the distance between the ground truth and a default box to find the best match between them for enhanced learning efficiency.

**Figure 3:**
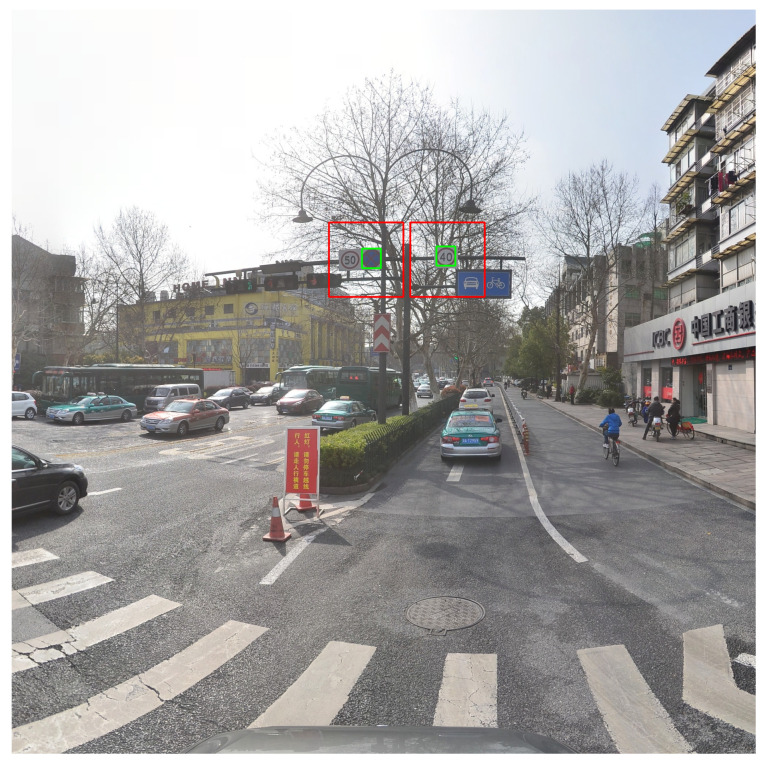
Improved matching with aligned matching strategy. The same color code is used as in Figure 1.

Figure 3 shows the results of using aligned matching for the cases shown in Figure 1. As shown in Figure 1, each ground truth object is off-center from the matched default box with a different aspect ratio. In contrast, as shown in Figure 3, for each ground truth box, aligned matching is able to find a default box with the same aspect ratio and the closest distance.

## 4. Evaluation

To evaluate the effectiveness of our aligned matching for small object detection, we first compared the performance of SSD with and without aligned matching on the Tsinghua-Tencent 100K [28], which is composed mostly of small objects. We also compared the performance of of SSD with and without aligned matching on the Pascal VOC 07 + 12 [31] to evaluate the performance of aligned matching on typical datasets. Additionally, we compared the performance of aligned matching to those of state-of-the-art detectors. In our experiments, we used a PyTorch implementation of SSD [29] and three SSD network models with different input sizes, SSD300, SSD512, and SSD1024. SSD1024 is extended from SSD512 by adding a convolutional layer conv13_2 to the end of extra feature layers of SSD512. For reproducibility, we used identical seeds and deterministic algorithms for all experiments. For the evaluation metric, we use average precision (AP) with an IoU threshold of 0.5 and mean average precision (mAP), which is the average of AP across all classes. All the experimental results were obtained using a system equipped with an Intel Xeon Silver 4210R CPU and NVIDIA GeForce RTX 3090 GPUs.

### 4.1. Performance Comparison with the Tsinghua-Tencent 100K Dataset

The Tsinghua-Tencent 100K (TT100K) comprises 16,787 annotated images of traffic signs with 221 classes. Because most classes in TT100K are associated with a relatively small number of images, the five classes (pn, pne, i5, p11, pl40) that appear the most frequently in TT100K were used in our experiments. Of a total 5569 images belonging to the five classes, 5125 were used for training and 444 for evaluation.

We trained SSD models with a batch size of 16 and used the Adam optimizer [32] with $\beta_{1}=0.9$, $\beta_{2}=0.999$, and $\epsilon =10^{-8}$ to perform training effectively. The batch size was set to 8 for SSD1024 due to memory restrictions. The learning rate was set to $10^{-4}$ for the first 50,000 iterations, $10^{-5}$ for the next 25,000 iterations, and $10^{-6}$ for the next 25,000 iterations. We ended the training at the 100,000th iteration. The smallest and largest default box sizes were set to 0.04 and 0.9 (i.e., the default box size is 4%/90% of the input image size), respectively. Unless otherwise specified, the largest default box scale used to obtain experimental results shown throughout this section was 0.9.

As shown in Table 2, SSD with an aligned matching strategy (denoted as Aligned) exhibited an 8% performance improvement over the original SSD matching strategy (denoted as Legacy) even for SSD300. The performance gap between the two became larger as the SSD’s input size increased, showing the greatest performance difference of 22% in the SSD1024 case. Note that SSD models with aligned matching always exhibited higher APs compared to their legacy counterparts for all classes. These results clearly demonstrate that aligned matching is very effective for small object detection, regardless of the input size of SSD.

**Figure 4:**
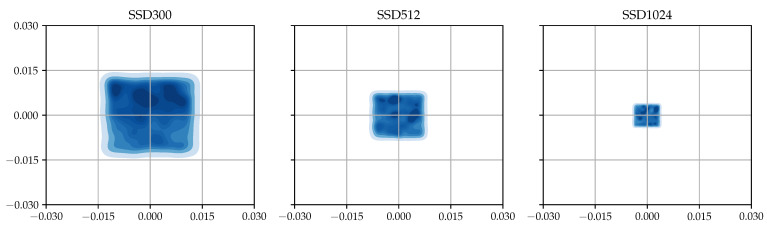
Relative center point distribution obtained from TT100K using aligned matching.

The performance advantage of aligned matching comes from the fact that the method can locate more ground truth objects at the receptive field’s center. Figure 4 shows the distribution of relative positions between each ground truth’s center point and the center point of the default box matched to the ground truth in the case of aligned matching. Compared with the original SSD case shown in Figure 2, we can see that aligned matching is able to choose the default boxes that are closer to the ground truth, that is, darker areas are located near the center. In the SSD300 case, the distribution difference is rather modest, but the difference becomes more evident as the input size increases, which is in accordance with the mAP results shown in Table 2.

### 4.2. Performance Comparison with Pascal VOC 07 + 12

The Pascal VOC dataset comprises images of everyday objects in 20 classes. The objects in the dataset were mostly larger than TT100K traffic signs. In our experiments, we used the common ’07 + 12’ set, that is, VOC 2007 trainval and VOC 2012 trainval were used for training, and VOC 2007 test was used for evaluation. We trained SSD models with a batch size of 32 (Due to memory restrictions, we set the batch size to 8 in the case of SSD1024.) and used SGD with 0.9 momentum and 0.0005 weight decay as in Ref. [4]. To address the gradient explosion problem, we adopted the learning rate warmup [33,34] at the beginning of training. During the first five epochs, the learning rate was linearly increased from zero to $10^{-3}$ as in Ref.  [4]. The smallest box size was set to 0.1/0.07/0.04 in the case of SSD300/SSD512/SSD1024.

It may be observed from Table 3 that the performance difference between the two matching strategies in the case of the Pascal VOC dataset is insignificant compared with the TT100K case. This is because unlike TT100K, Pascal VOC comprises images of objects of varying sizes. Small but consistent performance improvements with aligned matching can be observed when we compare APs of each class. These results show that aligned matching enhanced the performance of SSD regardless of the datasets used.

**Figure 5:**
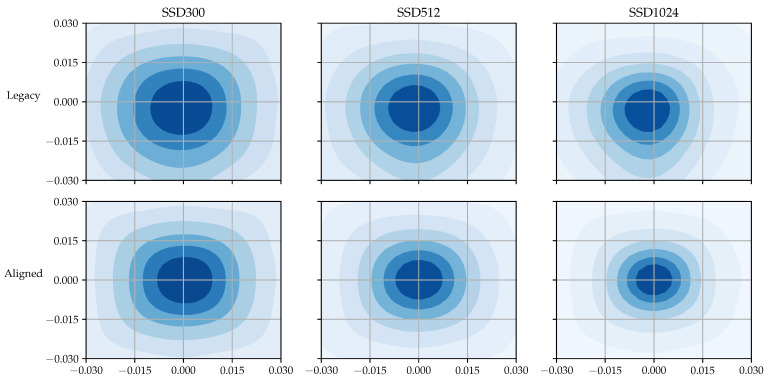
Relative center point distribution obtained from Pascal VOC.

The relative center point distribution heatmap for Pascal VOC dataset is shown in Figure 5. In accordance with the mAP results, the difference between the distributions for the two matching strategies is less noticeable with the Pascal VOC dataset compared to TT100K.

### 4.3. Performance Comparison with a Varying Default Box Size

Aligned matching exhibited significant performance improvements in small object detection tasks. These improvements were achieved by addressing the inefficiency of SSD when learning objects smaller than the minimum size of default boxes. As mentioned previously, optimizing the default box scales can also alleviate the problem. Optimizing the default box sizes, however, entails additional training efforts, i.e., one must repeat the same training as many times as the number of minimum default box sizes being considered. Here, we discuss the impact of the minimum default box size on the SSD’s performance with and without aligned matching. We compared the SSD’s performance with different minimum default box sizes (i.e., conv4_3 size) of 0.1, 0.07, and 0.04.

Table 4 shows the performance of SSD with varying minimum default box sizes. The table shows that the performance of SSD on TT100K increases as the minimum default box size decreases, regardless of the matching strategy used. In addition, aligned matching is always more accurate than legacy matching regardless of default box sizes, and the performance enhancement is rather dramatic, 3.6 times higher mAP, in the case of SSD1024 with 0.1 minimum default box size. More importantly, the performance gap between different minimum default box sizes is very large in legacy matching, e.g., 45.9% with SSD1024. In contrast, for aligned matching case, the gap was only 1.5% with SSD1024, which is significantly smaller than that in the legacy matching case.

**Figure 6:**
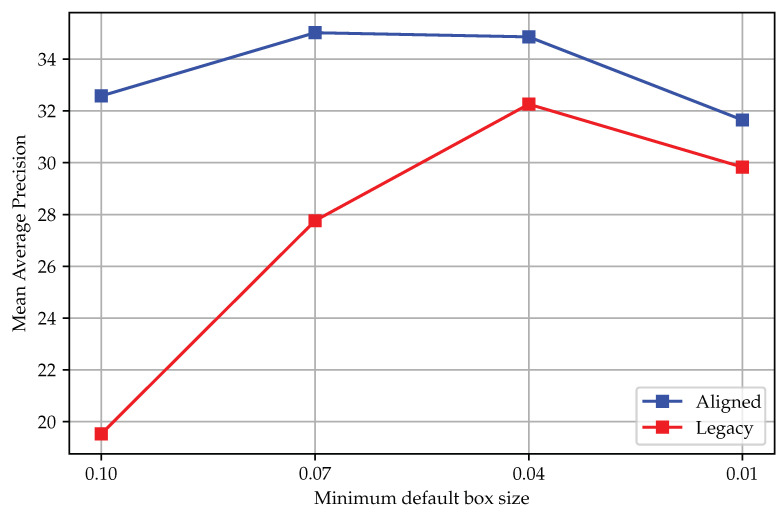
Performance of SSD300 with various default box sizes.

To check whether the performance trend of SSD with varying minimum default box sizes continued, we further compared the performance of SSD300 with two matching strategies as shown in Figure 6, with the minimum default box size varying from 0.1 to 0.01. The figure shows that the performance of both methods with the minimum default box size of 0.01 was lower than the case with 0.04, meaning that simply reducing the minimum default box size does not guarantee a better performance. Interestingly, the best legacy matching performance was on par with the worst aligned matching performance. As expected, aligned matching rendered SSD models more robust to the changes in the sizes of default boxes.

Table 5 shows the Pascal VOC results with various default box sizes. Similar to previous results, the differences in performance between aligned and legacy matching with the Pascal VOC dataset were minor compared to that with TT100K. More importantly, a performance fluctuation was also observed with the Pascal VOC dataset. This means that SSD might need optimization with respect to the default box scales, even for general object detection tasks.

### 4.4. Performance of SSD with K-Means Clustering

Similar problems can appear in other object detectors that match ground truth objects to default boxes based solely on IoU during training. You Only Look Once [3] (YOLO), another scheme that matches only with IoU, circumvents the problem by using K-means clustering to find the proper default box scale for a training set. However, as mentioned earlier, applying K-means to the training set involves a risk of overfitting because the distribution of object sizes in the training and test set can vary. Nevertheless, we evaluated the performance of SSD matching combined with K-means clustering, and compared it with that of aligned matching. In SSD with K-means clustering, objects in the training set were clustered based on their sizes and the default box size scale is determined based on the clusters’ centroids. For example, because SSD512 has seven feature maps, seven clusters were generated from the training set and the smallest and largest default box sizes were set as the centroids of the smallest and largest clusters, respectively.

Table 6 compares the performance of legacy matching combined with K-means clustering and aligned matching on TT100K dataset. As shown in the table, aligned matching outperformed legacy SSD with K-means in all cases, e.g., approximately a 11% point increase in mAP with SSD1024 model, meaning that K-means clustering was not as effective as aligned matching in solving problems in SSD’s matching strategy. By comparing the results to Table 2, it may be observed that SSD with K-means showed improvements over the case without K-means, e.g., an increase of approximately 3% increase in mAP with the SSD1024 model.

We also evaluated legacy matching with K-means on Pascal VOC to determine the effectiveness of the K-means technique on more general object detection tasks. In the evaluation, the Pascal VOC 07 + 12 set was used to train the models and to decide the scales of the default boxes using K-means clustering. From Table 7, it may be observed that aligned matching outperformed legacy matching with K-means, e.g., showing a 3.5% increase in mAP. The performance gap between the two methods increased with the size of the input, similar to TT100K. Note that legacy matching with K-means showed a performance decrease with SSD1024 compared to SSD512. Moreover, by comparing the results to Table 3, we can see that K-means clustering even degrades the performance of legacy matching. These results in fact reveal not only the inappropriateness of selecting the default box scales with K-means clustering but also the risk of degrading SSD’s performance when changing the default box scales. Obviously, aligned matching can avoid this risk and is more effective in solving problems in SSD’s matching strategy for both general and small object detection tasks.

### 4.5. Comparison with State-of-the-Art Detectors

Finally, we compared the performance of aligned matching to those of state-of-the-art detectors based on the results with the TT100K dataset reported in Ref. [18].

As shown in Table 8, aligned matching provided a significant performance boost on SSD1024, which sufficed to outperform the two-stage detectors, Faster R-CNN, Mask R-CNN, and FPN. For the classes i5, p11, and pl40, aligned matching outperformed all the two-stage detectors. In particular, in the case of pl40, aligned matching exhibited an AP of 80.1%, which was significantly better than that of FPN. Furthermore, the table shows that aligned matching was also more accurate than all single-stage detectors, including DSSD512, RFBNet512, ScratchDet, CAB Net, and CAB-s Net in terms of mAP. In fact, among the single-stage detectors, SSD1024 obtained not only the most accurate mAP but also the highest AP in the majority of classes, including pn, i5, and pl40. When the aligned matching strategy was used, SSD performed better than single-stage detectors specifically designed for small object detection. It is worth noting that CAB Net showed a lower inference speed compared to SSD [18], whereas aligned matching does not decrease SSD’s inference speed since it only affects SSD’s training process. To summarize, our aligned matching strategy enabled SSD to perform small object detection tasks more effectively such that it outperformed various state-of-the-art methods.

## 5. Conclusions

In this study, we have argued that the current IoU-based matching strategy of SSD could diminish its training efficiency when learning to detect small objects. This issue stems from the lack of a fallback option when multiple default boxes have the same IoU value. To address this issue, we have proposed an *aligned matching* strategy that considers the aspect ratio and center point distance in addition to IoU when determining the best match to ensure that the proper default box is selected even for small objects. The results of our experiments on the TT100K and Pascal VOC datasets showed that SSD with aligned matching achieved a significant improvement over the original SSD in small object detection without sacrificing performance on large objects, which was possible without requiring extra parameters or training. The performance improvement of SSD with aligned matching could be as high as an increase of 60% in terms of mAP. In addition, our results showed that SSD could surpass the performance of state-of-the-art detectors designed specifically for detecting small objects. The source code and trained weight files used in this study are available at https://github.com/ksh108405/aligned-matching (accessed on 26 January 2023).

## Figures and Tables

**Table 1:** Results of various SSD architectures on the small object dataset.

Model	mAP	AP				
		pn	pne	i5	p11	pl40
SSD300	19.5	31.3	21.4	17.4	12.2	15.3
SSD512	30.5	44.8	36.4	26.8	20.7	24.0
SSD1024	23.4	29.6	25.8	20.4	16.6	24.4

**Table 2:** SSD’s performance on TT100K.

Model	Matching Strategy	mAP	AP				
			pn	pne	i5	p11	pl40
SSD300	Legacy	32.3	44.2	44.7	38.3	11.2	22.8
	Aligned	34.9	46.9	47.8	42.2	11.7	23.3
SSD512	Legacy	53.3	67.4	59.3	58.5	38.7	42.4
	Aligned	62.9	77.9	70.9	68.6	46.4	50.8
SSD1024	Legacy	69.3	78.2	68.7	77.5	59.8	62.3
	Aligned	84.7	87.6	89.4	89.4	76.9	80.1

**Table 3:** SSD’s performance on Pascal VOC.

Model	Matching Strategy	mAP	AP																			
			Aero	Bike	Bird	Boat	Bottle	Bus	Car	Cat	Chair	Cow	Table	Dog	Horse	Mbike	Pers	Plant	Sheep	Sofa	Train	Tv
SSD300	Legacy	59.8	67.0	66.4	56.9	48.7	23.5	70.9	73.5	76.7	35.8	66.3	59.9	72.4	75.5	67.3	60.2	28.2	59.1	59.1	69.4	58.8
	Aligned	60.6	68.3	66.4	56.3	52.4	24.2	69.5	74.8	76.3	36.7	65.4	58.8	71.6	76.7	68.4	60.8	28.2	59.1	66.7	75.1	56.7
SSD512	Legacy	63.3	68.4	73.4	59.9	52.5	34.0	73.1	77.3	75.6	41.7	70.9	58.6	74.6	77.2	72.6	64.3	33.6	61.6	62.1	74.6	60.3
	Aligned	63.5	68.5	74.7	60.9	49.7	37.2	74.1	77.4	77.7	41.9	70.2	53.7	77.3	79.9	70.7	64.2	34.2	63.5	59.1	73.7	62.0
SSD1024	Legacy	64.2	74.1	75.3	62.2	50.5	45.1	73.9	81.7	74.4	43.6	70.5	57.9	67.8	75.3	70.3	70.0	36.6	64.0	57.6	72.5	59.9
	Aligned	64.6	72.8	74.5	61.0	53.6	47.3	72.4	81.9	73.4	44.0	70.7	56.9	68.8	79.5	72.4	70.5	35.6	65.5	58.7	70.3	62.3

**Table 4:** SSD’s performance on TT100K with various default box sizes.

Model	Matching Strategy	Minimum Default Box Size		
		0.1	0.07	0.04
SSD300	Legacy	19.5	27.8	32.3
	Aligned	32.6	35.0	34.9
SSD512	Legacy	30.5	51.8	53.3
	Aligned	59.3	60.3	62.9
SSD1024	Legacy	23.4	63.7	69.3
	Aligned	83.2	84.6	84.7

**Table 5:** Performance on Pascal VOC with various default box sizes.

Model	Matching Strategy	Minimum Default Box Size		
		0.1	0.07	0.04
SSD300	Legacy	59.8	58.1	55.4
	Aligned	60.6	57.9	56.0
SSD512	Legacy	62.4	63.3	62.1
	Aligned	62.7	63.5	61.8
SSD1024	Legacy	57.0	62.1	64.2
	Aligned	57.5	61.9	64.6

**Table 6:** SSD’s performance on TT100K with K-means clustering.

Model	Method	mAP	AP				
			pn	pne	i5	p11	pl40
SSD300	K-Means	27.9	41.1	37.1	35.8	8.2	17.4
	Aligned	34.9	46.9	47.8	42.2	11.7	23.3
SSD512	K-Means	48.3	57.1	58.4	60.0	38.4	27.7
	Aligned	62.9	77.9	70.9	68.6	46.4	50.8
SSD1024	K-Means	72.1	77.3	78.2	78.5	57.6	68.8
	Aligned	84.7	87.6	89.4	89.4	76.9	80.1

**Table 7:** SSD’s performance on Pascal VOC with K-means clustering.

Model	Method	mAP	AP																			
			Aero	Bike	Bird	Boat	Bottle	Bus	Car	Cat	Chair	Cow	Table	Dog	Horse	Mbike	Pers	Plant	Sheep	Sofa	Train	Tv
SSD300	K-Means	60.2	68.9	67.2	54.8	44.2	25.3	71.5	74.3	75.2	38.0	64.9	57.1	72.2	75.6	70.5	61.5	29.7	59.3	63.6	72.7	56.8
	Aligned	60.6	68.3	66.4	56.3	52.4	24.2	69.5	74.8	76.3	36.7	65.4	58.8	71.6	76.7	68.4	60.8	28.2	59.1	66.7	75.1	56.7
SSD512	K-Means	62.5	71.1	72.9	59.5	49.9	34.8	72.1	76.6	76.1	40.3	68.3	56.0	73.4	75.5	69.1	64.2	32.4	60.4	59.7	73.8	63.2
	Aligned	63.5	68.5	74.7	60.9	49.7	37.2	74.1	77.4	77.7	41.9	70.2	53.7	77.3	79.9	70.7	64.2	34.2	63.5	59.1	73.7	62.0
SSD1024	K-Means	61.1	65.6	72.3	57.4	39.0	42.6	71.9	77.5	72.6	45.1	63.7	59.4	66.1	71.9	69.0	67.3	31.7	58.6	58.0	71.6	60.5
	Aligned	64.6	72.8	74.5	61.0	53.6	47.3	72.4	81.9	73.4	44.0	70.7	56.9	68.8	79.5	72.4	70.5	35.6	65.5	58.7	70.3	62.3

**Table 8:** Comparison of aligned matching with state-of-the-art detectors.

Method	Backbone	mAP	AP				
			pn	pne	i5	p11	pl40
Faster R-CNN [2]	VGG16	43.8	37	47	45	38	52
Faster R-CNN [17]	ResNet-50	72.7	77.8	87.5	79.7	58.5	60.2
FPN [12]	ResNet-101	82.9	**89.0**	89.8	88.3	75.9	71.5
Mask R-CNN [5]	ResNet-101	82.6	88.6	**90.5 **	89.0	76.9	68.2
DSSD512 [16]	ResNet-101	73.6	67.2	88.9	88.6	55.1	68.2
RFBNet512 [35]	VGG16	78.5	78.0	88.2	87.9	66.9	71.6
ScratchDet [36]	ResNet-101	78.8	76.7	89.4	89.2	65.3	73.2
CAB Net [18]	VGG16	83.3	85.4	89.5	**89.4 **	**77.6 **	74.8
CAB-s Net [18]	VGG16	81.7	82.2	89.2	**89.4 **	72.4	75.3
SSD1024 w/aligned matching	VGG16	**84.7**	87.6	89.4	**89.4**	76.9	**80.1**

## Data Availability

The source code and trained weight files used in this study are available at the GitHub repository (https://github.com/ksh108405/aligned-matching) (accessed on 26 January 2023).
